# Multiple roles for Vitamin B_6_ in plant acclimation to UV-B

**DOI:** 10.1038/s41598-018-38053-w

**Published:** 2019-02-04

**Authors:** Gyula Czégény, László Kőrösi, Åke Strid, Éva Hideg

**Affiliations:** 1 Department of Plant Biology, University of Pécs, Pécs, Hungary; 2 Research Institute for Viticulture and Oenology, University of Pécs, Pécs, Hungary; 3 School of Science & Technology, Örebro Life Science Center, Örebro University, Örebro, Sweden

## Abstract

Direct and indirect roles of vitamin B_6_ in leaf acclimation to supplementary UV-B radiation are shown in vitamin B_6_ deficient *Arabidopsis thaliana* mutant *rsr4-1* and C24 wild type. Responses to 4 days of 3.9 kJ m^−2^ d^−1^ biologically effective UV-B dose were compared in terms of leaf photochemistry, vitamer content, and antioxidant enzyme activities; complemented with a comprehensive study of vitamer ROS scavenging capacities. Under UV-B, *rsr4-1* leaves lost more (34%) photochemical yield than C24 plants (24%). In the absence of UV-B, *rsr4-1* leaves contained markedly less pyridoxal-5′-phosphate (PLP) than C24 ones, but levels increased up to the C24 contents in response to UV-B. Activities of class-III ascorbate and glutathione peroxidases increased in C24 leaves upon the UV-B treatment but not in the *rsr4-1* mutant. SOD activities remained the same in C24 but decreased by more than 50% in *rsr4-1* under UV-B. Although PLP was shown to be an excellent antioxidant *in vitro*, our results suggest that the UV-B protective role of B_6_ vitamers is realized indirectly, via supporting peroxidase defence rather than by direct ROS scavenging. We hypothesize that the two defence pathways are linked through the PLP-dependent biosynthesis of cystein and heme, affecting peroxidases.

## Introduction

Vitamin B_6_ (pyridoxine, PN) and its vitamer derivatives pyridoxal (PL), pyridoxamine (PM) and its phosphorylated analogues) have dual roles in plants. They are important for both development^1–4^ and stress tolerance^1,3,5–9^. The role in development is likely to be due to the fact that the pyridoxine vitamer pyridoxal 5′-phosphate (PLP) is a crucial co-factor of a range of enzymes important for biosynthesis of building blocks of biological macromolecules^10^. On the other hand, one vital mechanism behind the stress tolerance conferred by the pyridoxine vitamers is their ability to function as quenchers of reactive oxygen species^7,11–15^.

Vitamin B_6_, primarily PLP, is in plants, fungi and most eubacteria synthesized from ribose 5-phosphate, glyceraldehyde 3-phosphate and glutamine by a large 24 polypeptide multisubunit complex (as revealed from studies of the *Bacillus subtilis* enzyme) that consists of 12 units of the PDX1 synthase protein and 12 units of the PDX2 glutaminase protein^16^. In plants, there is one gene encoding PDX2, the mutation of which is lethal, and three PDX1 genes (*PDX1*.*1*-*PDX1*.*3*). PDX1.1 and PDX1.3 are enzymatically functional proteins. PDX1.2 on the other hand is not catalytically active. Instead, this protein has a regulatory role on vitamer biosynthesis through interaction with the multisubunit enzyme, primarily during stress^8,9^. Environmental factors that have been shown to cause stress in *A*. *thaliana* plants that have been mutated in genes of one of the PDX1 subunits include salt and osmotic stress^1,5^, high light and photo-oxidative stress^3,15^, heat^9,14^, and ultraviolet-B light (UV-B, 280–315 nm)^6,7^.

UV-B is part of the radiation spectrum of the sun that plants are exposed to and dependent on. UV-B is generally a morphological factor under normal conditions^17–19^ but can be a stressor in extreme environments or unusual circumstances^20,21^, or when plants are exposed to multiple stresses at the same time^22^. UV-B is generally sensed by plants through the UV RESISTANCE LOCUS 8 (UVR8) photoreceptor and its downstream signaling components^19^ which in turn regulate over 100 genes. The gene encoding the PDX1.3 protein is one of these and is up-regulated by UV-B^23–25^. Also, UV-B exposure leads to increased levels of PDX1 protein in Arabidopsis^6,26,27^ and increased levels of vitamin B_6_^6^. In fact, one of the more non-specific modes of action of UV-B on plants is the formation of ROS^28^ and the UV-B-induced increase in vitamin B_6_ content in plants most likely is a result of increased oxidative pressure. This oxidative pressure was shown to increase in an *A*. *thaliana pdx1*.*3* mutant^7^ that still had a functional *PDX1*.*1* gene.

In order to further elucidate the roles of the different pyridoxine vitamers *in planta*, C24 wild type and *rsr4-1* mutant *A*. *thaliana* were used to draw conclusions about the roles that ROS and the pyridoxine vitamers play during UV-B exposure. Created from C24 using the mutagenic alkylating agent ethyl-methanesulfonate, a glabrous, inbred segregate mutant of *A*. *thaliana*, was shown to have a non-functional PDX1.3 protein with respect to vitamin B_6_ biosynthesis due to a point mutation in one of the *PDX1* genes^2^.

Several publications confirm high ROS reactivities of B_6_ vitamers. In computational studies Matxain *et al*.^12–14^ showed that the basic vitamer pyridoxine (PN) could function as a substrate for attack by a number of different ROS (^•^OH, hydroxyl radical; ^•^OOH, perhydroxyl radical; and ^1^O_2_, singlet oxygen) and thereby as antioxidant for these chemical species. The ^1^O_2_ reactivity of PN was shown experimentally by Bilski *et al*.^29^, who also found pyridoxamine (PM) and pyridoxal (PL) better ^1^O_2_ quenchers than PN or PLP in D_2_O (deuterium oxide). Although theoretical studies predicted that PN was not reactive toward the superoxide anion radical (O_2_^•−^;^13^), Denslow *et al.*^30^ presented experimental evidence for the superoxide neutralizing capacity of PN *in vitro*, and also showed the reactivity of PL, and to a smaller extent of pyridoxamine PM, to this ROS. In order to present a comprehensive picture of their potential antioxidant functions *in planta*, we compared reactivities of PN, PL, PM and PLP to all four major ROS (O_2_^•−^, H_2_O_2_, ^•^OH and ^1^O_2_) *in vitro*.

Based on the UV sensitivity of the *pdx1*.*3* mutant^7^, we hypothesized that *rsr4-1* plants (i) will either be more sensitive to supplementary UV-B doses than the C24 wild type, (ii) or compensate for presumably less efficient non-enzymatic ROS scavenging by higher antioxidant enzyme activities. Our results supported the first scenario and suggested an indirect effect by B_6_ vitamers in successful stress acclimation via enzymatic defence.

## Results and Discussion

### Photosynthetic responses of *A*. *thaliana* leaves to supplemental UV-B

In the absence of UV treatment, C24 and *rsr4-1* leaves displayed similar photochemical efficiencies. At 5 weeks of age, mutant leaves showed no visible signs of previously described leaf yellowing^2^ (data not shown), and are rather in line with reports on normal development of vitamin B_6_ deficient plants^5,15^. Accordingly, we observed no difference between maximum PSII efficiencies of the two genotypes assessed as Fv/Fm (maximum PSII quantum yield). Moreover, the *rsr4-1* mutant displayed even slightly higher light acclimated PSII photochemical yield (ϕPSII) than the wild type C24 when tested using 55 μmol m^−2^ s^−1^ blue actinic light. This results in 8–9% lower regulated and non-regulated non-photochemical quenching yields, Y(NPQ) and Y(NO), respectively, in *rsr4-1* than in C24 leaves (Table 1). Higher non-photochemical quenching was reported for another vitamin B_6_ deficient mutant *pdx1* and was explained by a more efficient photoconversion of violaxanthin to zeaxanthin in the mutant than in the corresponding wild type^15^, although the authors made this observation at high light conditions only, i.e. at 5–20-times higher photosynthetically active radiation (PAR) than was applied in our experiment. The opposite finding in our experiment, i.e. a lower non-photochemical quenching yields in the *rsr4-1* mutant at lower PAR, suggests that the mutant does not sense and respond to the lower PAR as a potential photo-oxidative stress.

**Table 1:** Effects of supplemental UV radiation on maximum (Fv/Fm) and 55 μmol m^−2^ s^−1^ PAR acclimated effective (ϕPSII) quantum yields, the regulated (Y(NPQ)) and non-regulated (Y(NO)) non-photochemical quenching of PSII (Materials and Methods). Data are presented as means ± SD.

	Fv/Fm	ϕPSII	Y(NPQ)	Y(NO)
C24 C	0.852 ± 0.005	0.655 ± 0.015	0.123 ± 0.005	0.221 ± 0.019
C24 UV	0.659 ± 0.054*	0.499 ± 0.051*	0.123 ± 0.006	0.378 ± 0.051*
*rsr4-1* C	0.853 ± 0.003	0.658 ± 0.014^#^	0.114 ± 0.005^#^	0.201 ± 0.011^#^
*rsr4-1* UV	0.574 ± 0.050*^#^	0.437 ± 0.050*^#^	0.124 ± 0.007*	0.440 ± 0.047*^#^

*UV effect: significant difference between control (C) and UV-exposed (UV) leaves (*p* < 0.05, n = 8) of the same genotype.

^#^Genotype effect: significant difference between wild type (C24) and B_6_ mutant (*rsr4-1*) control leaves (*p* < 0.05, n = 8) under the same irradiation conditions.

Supplemental UV imposed a mild stress in both genotypes, as indicated by lower maximum (Fv/Fm) and light acclimated (ϕPSII) photochemical yields than in controls (Table 1). The UV-induced loss was higher (ca 32%) in the *rsr4-1* mutant than in the C24 wild type (ca 22%). Yields of photochemical energy conversion in PSII were lowered either entirely (C24 plants) or mostly (*rsr4-1* plants) at the expense of increasing energy dissipation via non-regulated non-photochemical quenching Y(NO), signifying suboptimal capacities of photoprotective reactions, that may lead to photodamage^31^. The UV treatment had no effect on the quantum yield of regulated non-photochemical quenching Y(NPQ) in C24 leaves but resulted in a 9% increase in the *rsr4-1* mutant. The latter change brought up Y(NPQ) in UV-treated *rsr4-1* to values similar to those in C24. Such an increase indicates elevation of defence against photo-oxidative stress at the PSII level in the mutant that is not occurring in the wild type. This might be explained by the above-mentioned xanthophyll-cycle related changes observed in the *pdx1* mutant under high light stress^15^. However, this pathway is probably inefficient in the *rsr4-1*, as indicated by the relatively small extent of its increase compared to that of Y(NO). Although the biochemical explanation of non-regulated non-photochemical pathways is still incomplete, it is generally agreed that some of the multiple pathways behind Y(NO), especially those driving this parameter above 0.2–0.25 in light acclimated leaves, reflect the inability of a plant to protect itself against damage caused by excess illumination^31,32^, presumably via increased ROS production. In the following, we examined how the antioxidant systems of C24 and *rsr4-1* leaves met this challenge.

### Vitamin B_6_ content of *A*. *thaliana* leaves, and *in vitro* ROS neutralizing potential of these compounds

The three basic vitamers (PL, PM, PN) and the activated form of vitamin B_6_ (PLP) were quantified in C24 wild type and *rsr4-1* mutant leaves using HPLC. PN contents were low in both genotypes, representing only 1–2% of the total, and showing that PN is the least common B_6_ vitamer in both genotypes (Table 2). In C24 leaves, the amounts of PL and PLP were the highest, while PLP concentration in the mutant were below the one in the wild type (Table 2). Our data are in agreement with those of Wagner *et al*.^2^ that established a PL > PM > PN order of the amounts of these compounds in both genotypes. They also found a higher PL/PM ratio in C24 than in *rsr4-1* leaves, although their absolute concentrations were different from ours, most likely due to differences in growth conditions and extraction procedures. Our observations do not agree with Havaux *et al*.^15^ who found high PN and PM, and low PL concentrations. However, these authors analysed chloroplasts only (as opposed to whole leaf extracts in our case) and used a different *Arabidopsis* wild type, Col-0. The UV treatment applied led to elevated concentrations of all vitamers in mutant leaves but did not affect the B_6_ content in the wild type. The UV treatment brought the PLP levels in *rsr4-1* leaves up to those in C24 under UV and increased the PL and PM contents of the mutant to 30% higher than those in the wild type (Table 2).

**Table 2:** B_6_ vitamer profiles of *A*. *thaliana* leaves. Means ± SD are expressed as ng vitamer mg^−1^ leaf FW.

	pyridoxal (PL)	pyridoxamine (PM)	pyridoxine (PN)	pyridoxal 5′-phosphate (PLP)
C24 C	1.048 ± 0.132	0.694 ± 0.060	0.064 ± 0.037	1.357 ± 0.277
C24 UV	1.160 ± 0.177	0.690 ± 0.138	0.052 ± 0.015	1.184 ± 0.251
*rsr4-1* C	1.117 ± 0.147	0.704 ± 0.070	0.030 ± 0.008	0.797 ± 0.207^#^
*rsr4-1* UV	1.530 ± 0.141*^#^	0.895 ± 0.103*^#^	0.062 ± 0.009*	1.088 ± 0.164*

*UV effect: significant difference between control (C) and UV-exposed (UV) leaves (*p* < 0.05, n = 8) of the same genotype.

^#^Genotype effect: significant difference between wild type (C24) and B_6_ mutant (*rsr4-1*) control leaves (*p* < 0.05, n = 8) under the same irradiation conditions.

*In vitro* analyses of vitamer reactivities toward ROS provide an excellent tool to study their antioxidant potential. However, the realization of these studies *in planta* depends on a number of factors, primarily on vitamer localization. Several studies of vitamin B_6_ quenching of ROS have previously been performed^12–14,29,30,33^ but these investigations either were limited to one or two ROS only or involved fewer forms of vitamin B_6_ than the present work. Thus, to our best knowledge, the results in Table 3 provide the first comprehensive data set of antioxidant capacities of four B_6_ vitamers (the three basic forms PL, PM, PN, and the activated form PLP) against the four principal ROS: singlet oxygen (^1^O_2_), superoxide anion (O_2_^•−^), hydrogen peroxide (H_2_O_2_) and hydroxyl radical (^•^OH). The only previous study including the antioxidant capacities of the same four vitamers established the following series of total (physical and chemical) ^1^O_2_ quenching rates in D_2_O: PN < PLP < PL = PM, with a 35% difference between the lowest and the highest efficiency (Bilski *et al*., 2000). Our data confirm the strong reactivity of PM to ^1^O_2_ but we found no detectable reactivity for PLP for this particular ROS, and much lower reactivities for PN and PL than for PM. These discrepancies may be due to differences in the solvents and experimental conditions used in the two different studies. In support of the theoretical result of Matxain *et al*.^13^, PN showed one or two orders of magnitude lower reactivity to O_2_^•−^ than to the other ROS in the present study. Furthermore, our comprehensive examination showed that PN is an inefficient O_2_^•−^ scavenger (Table 3).

**Table 3:** ROS-specific neutralizing capacities of B_6_ vitamers.

	anti-^1^O_2_	anti-O_2_^•−^	anti-H_2_O_2_	anti-^•^OH
pyridoxal (PL)	69.81 ± 6.84	14.47 ± 5.30	25.04 ± 2.78	42.71 ± 0.54
pyridoxamine (PM)	459.7 ± 52.19	0.5 ± 0.09	39.69 ± 7.20	16.21 ± 0.58
pyridoxine (PN)	5.69 ± 0.06	0.47 ± 0.06	48.17 ± 10.09	8.29 ± 0.65
pyridoxal 5′-phosphate (PLP)	ND	1.04 ± 0.02	287.90 ± 64.81	66.71 ± 4.45

Singlet oxygen (^1^O_2_), superoxide anion (O_2_^•−^), hydrogen peroxide (H_2_O_2_) and hydroxyl radical (^•^OH) antioxidant abilities were expressed as µM vitamer/µM Trolox equivalents. ND, non-detectable. Data represent means ± SD (n = 6–8).

Based on a comparison of leaf B_6_ vitamer profiles (Table 2) and *in vitro* vitamer ROS reactivities (Table 3), the observed UV-induced increase in PM, PL and PLP contents in *rsr4-1* leaves may support defence against H_2_O_2_, ^•^OH, and ^1^O_2_. Singlet oxygen in leaves is mainly produced in photodynamic energy transfer from excited PSII chlorophylls to oxygen^34^, and defence against this ROS is realized non-enzymatically only. There was no significant change in the highly ^1^O_2_-reactive PM content in C24 plants in response to UV, which is in line with the earlier observation that UV-B had little effect on energy transfer derived ^1^O_2_ concentrations in spinach leaves^35^. In contrast, the higher PM content in *rsr4-1* leaves under PAR plus UV-B than under PAR only suggests a response to photo-oxidative stress and argues against the role of UV-inducible high Y(NPQ) (Table 1) in preventing ^1^O_2_ production. Interestingly, the amount of PLP, the most capable vitamer of providing H_2_O_2_ and ^•^OH neutralization (Table 3) did not increase in the C24 wild type upon UV-exposure (Table 2), although the defence against these two ROS was found pivotal in the UV acclimation of tobacco leaves^36,37^.

### Leaf antioxidant responses to supplemental UV-B

In the following, we compare the activities of enzymes neutralizing electron transfer derived ROS (H_2_O_2_ and O_2_^•−^) in leaves of wild type and mutant plants and discuss their UV-B-induced changes. Because ^•^OH neutralization is only supported non-enzymatically, this was also included in the analysis.

**Figure 1:**
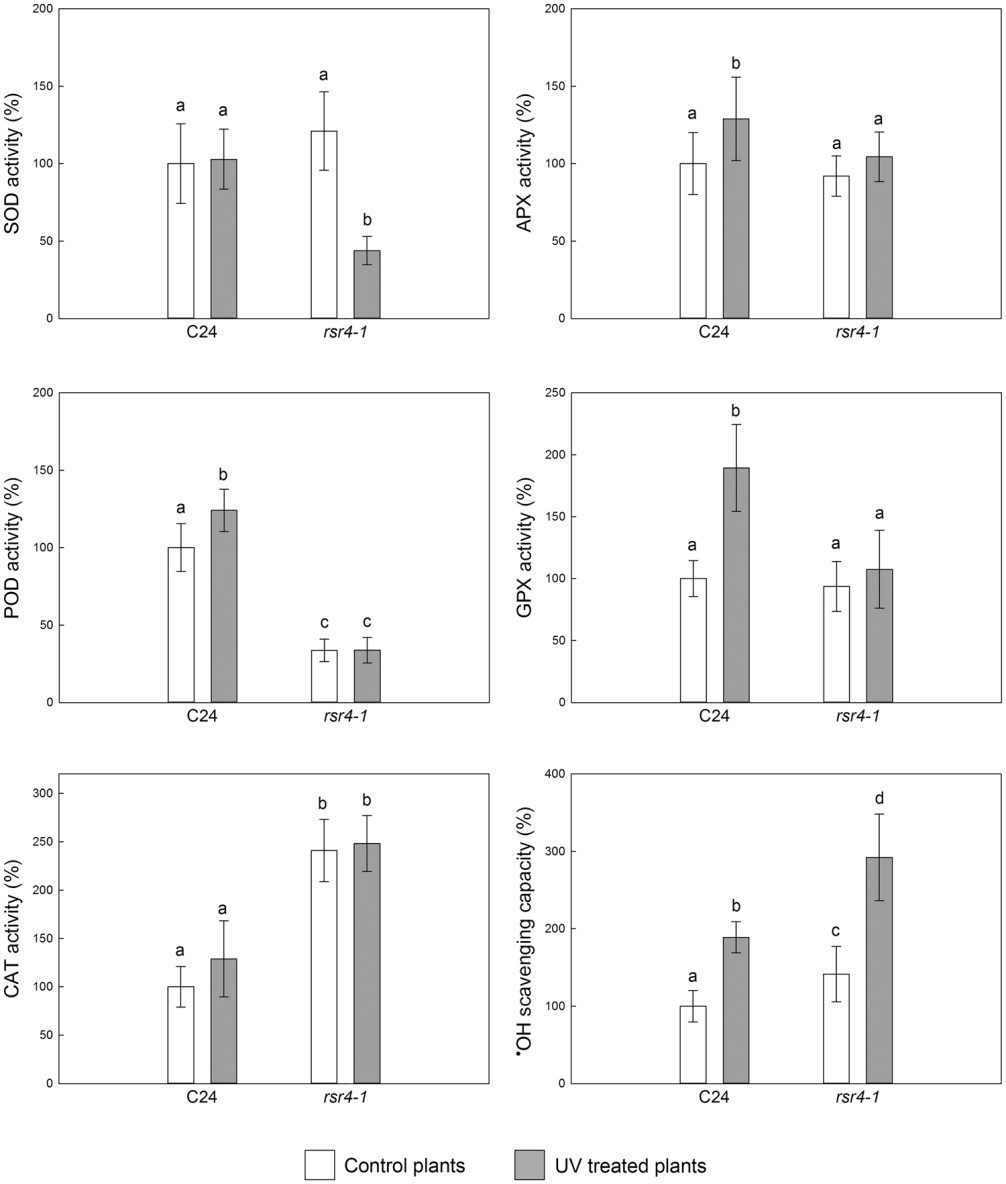
UV-B-induced alteration of antioxidant enzyme activities and hydroxyl radical scavenging capacities of *A*. *thaliana* leaves. ‘C24’, wild type *A*. *thaliana*; *rsr4-1*, *A*. *thaliana* mutant reduced in vitamin B_6_ synthesis. Control plants were exposed to PAR only and UV-treated plants were exposed to PAR and supplemental UV radiation. White bars represent data from plants kept under PAR only and grey bars correspond to data measured in UV-treated plants. Data are expressed as % of control C24 averages. Bar lengths correspond to means and error bars represent standard deviations (n = 8). For each antioxidant, the four means were compared pair wise and significantly (*p* < 0.05) different means are marked with different letters. 100% SOD = 37.17 ± 9.2 U mg^−1^ protein, 100% APX = 6.09 ± 1.19 U mg^−1^ protein, 100% CAT = 5.41 ± 1.14 U mg^−1^ protein, 100%, GPX = 80.3 ± 11.6 U g^−1^ protein, 100% POD = 231.79 ± 35.9 U mg^−1^ protein, 100% hydroxyl radical (^•^OH) scavenging = 17.45 ± 3.53 µM Trolox equivalent g^−1^ fresh leaf weight.

Under growth light conditions, in the absence of UV, the *rsr4-1* mutant controlled cellular H_2_O_2_ concentrations by significantly higher catalase (CAT, EC 1.11.1.6) activity, while keeping class III peroxidase (POD, EC 1.11.1.7) activity lower than the wild type C24 plants. There were no significant differences between the two genotypes in superoxide dismutase (SOD, EC 1.15.1.1), ascorbate peroxidase (APX, EC 1.11.1.11), or glutathione peroxidase (GPX, EC 1.11.1.9) activities (Fig. 1). In response to the UV treatment, POD, APX and GPX activities increased in wild type C24 leaves whereas SOD and CAT remained unchanged. Unchanged SOD and increased peroxidase activities under UV are in line with our observation of higher activation of APX and POD than of SOD in tobacco leaves^36,37^. This strategy keeps cellular H_2_O_2_ concentrations low in order to decrease the risk of oxidative damage^38^, potentially aggravated by the UV-B photo-conversion of H_2_O_2_ to ^•^OH^7^. The increased ^•^OH scavenging capacity in C24 leaves under UV-B (Fig. 1), that was also found in tobacco leaves^36,37^, may serve as a second line of defence. In contrast, *rsr4-1* leaves were unable to up-regulate any peroxidase (GPX, APX or POD) activities upon the UV treatment. The observed strong decrease in SOD activity of UV-treated *rsr4-1* plants lowers the production of H_2_O_2_ via O_2_^•−^ dismutation and highlights the lack of efficient direct H_2_O_2_ scavenging in the mutant.

The lack of change in CAT activity in UV-irradiated C24 plants (Fig. 1) suggests that peroxisomal H_2_O_2_ production is either low or well regulated already and does not initiate oxidative stress. Although UV irradiation of wheat under different experimental conditions upregulated CAT^39,40^, we found no significant change in CAT activity in UV experiments with tobacco leaves either (Czégény *et al*., unpublished). Our results may be explained by assuming (i) a low photorespiratory activity in wild type plants grown under relatively low PAR in our growth chambers, and/or (ii) catalase reactions being unaffected by the applied UV treatment. Either hypothesis is in line with the lack of CAT UV response in the *rsr4-1* mutant (Fig. 1).

A weak line of enzymatic H_2_O_2_ neutralization explains the need for more efficient ^•^OH scavenging in the vitamin B_6_ mutant. Accordingly, non-treated *rsr4-1* plants had 40% more efficient ^•^OH neutralizing capacity than the wild type and both genotypes were capable of upregulating this activity under UV. Although B_6_ vitamers, especially PLP, are efficient ^•^OH antioxidants *in vitro* (Table 3), differences in leaf ^•^OH antioxidant capacity do not match the vitamin B_6_ content, and thus a strong contribution of other antioxidants has to be assumed. Potential other ^•^OH antioxidants include α-tocopherol, phenolic compounds, ascorbate and reduced glutathione (GSH)^41–44^, each shown to increase under mild UV-B exposure^45^. Among the above candidates, both the plant phenol chlorogenic acid and α-tocopherol were found to be more reactive to ^•^OH than ascorbate or GSH^43^. Chlorogenic acid is synthesized in *A*. *thaliana*^46^ and, similarly to other phenolic compounds, its synthesis is regulated by the UVR8 photoreceptor^47^.

**Figure 2:**
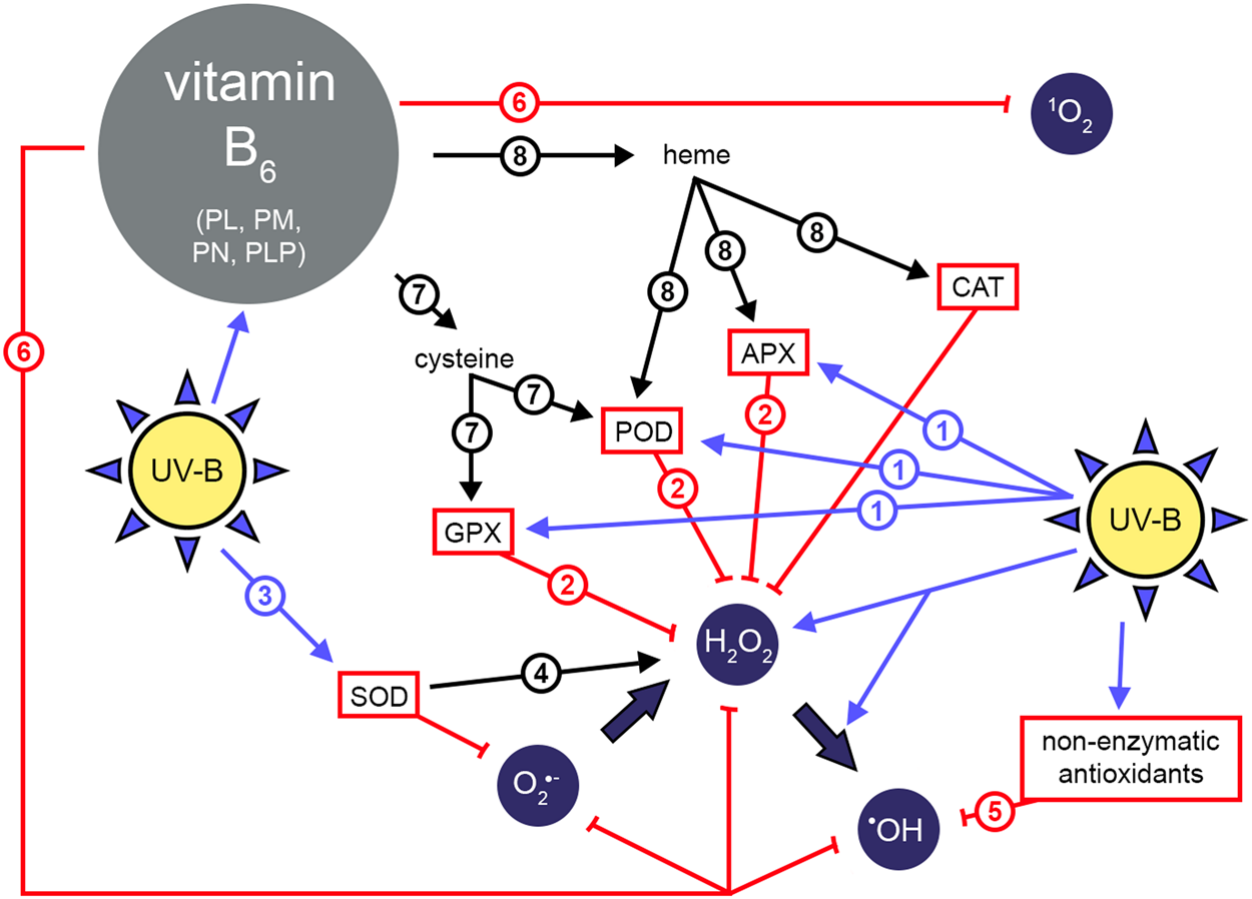
A schematic representation of pathways discussed in the present work. UV-B inducible and antioxidative routes are shown in blue and red, respectively. Routes are discussed in the text according to the encircled identifying numbers.

Figure 2 is a schematic representation of a hypothesis on possible roles of B_6_ vitamers in UV-B responses. Experiments with the C24 wild type in this work, just as our earlier studies using tobacco plants^36,37^, showed that supplemental UV-B enhanced the peroxidase defence (route-1 in Fig. 2), decreasing cellular H_2_O_2_ concentrations (route-2). Depending on the applied UV dose, SOD activities may also increase (route-3), although generally to a smaller extent than peroxidases^38^ in order to avoid excessive H_2_O_2_ production (route-4). Hydroxyl radical concentrations are kept low by the non-enzymatic antioxidants discussed above, including GSH (route-5). The B_6_ vitamers, PLP and PL, are also reactive toward a variety of ROS (Table 3, route-6 in Fig. 2).

Being a coenzyme, PLP takes part in several biosynthetic pathways, for example in cysteine biosynthesis. The PLP dependence of this pathway is in the conversion of *O*-acetylserine into cysteine^48^. Since both POD and GPX contain cysteine residues in the active site^49,50^, a nearly 50% lower PLP content in the *rsr4-1* mutant (Table 2) may contribute to the limited UV-B inducibility of these peroxidases (Fig. 1, route-7 in Fig. 2). Heme biosynthesis is also PLP dependent^51^ and thus the observed lower PLP content in the *rsr4-1* mutant may affect heme peroxidases, too (route-8 in Fig. 2). We hypothesise that the limited availability of both heme and cysteine in the *rsr4-1* mutant contributes to the low base level of POD and the lack of UV-B response of this enzyme. Although leaf PLP contents increased upon UV-B treatment in the *rsr4-1* mutant, additional levels do not seem to cover the multiplicity of pathways that are involved in acclimative responses to UV-B. Lower availability of cysteine implies lower GSH levels in vitamin B_6_ deficient mutants than in the wild type and may also limit the availability of phytochelatin in *rsr4-1* leaves. The latter increases the probability of H_2_O_2_ → ^•^OH conversion catalyzed by free iron^52^, a condition towards which the *rsr4-1* mutant responds with higher ^•^OH scavenging capacity than the wild type even in the absence of UV-B (Fig. 1). Elements of the above hypothesis, such as routes-7 and -8 are yet to be proven experimentally.

In summary, a comprehensive study of antioxidant responses to supplemental UV-B radiation in the *rsr4-1* mutant and the C24 wild type point to a multiple role of B_6_ vitamers in UV-B tolerance. Moreover, our results suggest a link between the UV-B sensitivity of vitamin B_6_ deficient plants that we identified in the *pdx1*.*3* mutant^6,7^ and the pivotal role of efficient H_2_O_2_ neutralizing in acclimation to UV-B^37,38^.

## Methods

### Plant growth and UV treatment

*A*. *thaliana* plants (C24 wild type and *rsr4-1* mutant) were grown using 90 μmol m^−2^ s^−1^ photosynthetically active radiation (PAR) in growth chambers with constant 70% relative humidity and 6 h/18 h, 22 °C/18 °C day/night conditions. 5-week old plants were divided into two groups, each containing eight plants from each genotype. The first group (UV plants) was exposed to supplemental UV radiation from Q-Panel UVB-313EL tubes (Q-Lab Ltd., Bolton, UK) through a cellulose diacetate filter (Courtaulds Chemicals, Derby, UK) between 10.00 and 14.00 daily for 4 days. The UV spectrum was centred around 318 nm. UV-B irradiation corresponded to 3.9 kJ m^−2^ d^−1^ biologically effective dose^53^. The second group (control plants) were kept under PAR only. Photosynthesis measurements were carried out at the end of the 4-day treatments, and then leaves were frozen in liquid N_2_ and stored at −80 °C for analytical measurements.

### Chlorophyll fluorescence measurements

Photosynthesis was characterized by chlorophyll fluorescence measurements using the MAXI version of Walz Imaging PAM. Maximum quantum yield of photosystem II (Fv/Fm) was measured after 30 min dark adaptation. Light acclimated effective PSII quantum yield (ϕPSII), regulated non-photochemical quenching (Y(NPQ)) and non-regulated non-photochemical quenching (Y(NO)) were determined under 55 μmol m^−2^ s^−1^ blue actinic light according to Klughammer & Schreiber^31^. In this model, the three PSII quantum yields are complementary as ϕPSII + Y(NPQ) + Y(NO) = 1, representing three possible pathways of disposing quanta.

### HPLC analysis

Thirty mg of leaves were ground in liquid nitrogen, placed into a plastic tube and extracted with 1.0 ml of 50 mM H_3_PO_4_ solution using ultrasonic bath for 15 min. The resulting suspension was centrifuged at 20,660 × g. Supernatants were filtered using a 0.22 μm PTFE syringe filter and analysed by high-performance liquid chromatography (HPLC). HPLC-FLD analysis was performed using a PerkinElmer Series 200 HPLC system consisting of a vacuum degassing unit, quaternary pump, autosampler, column thermostat and a fluorescence detector (FLD). Separations were achieved by using a Phenomenex Synergi^TM^ 4 µm Hydro-RP 80 Å, 250 × 4.6 mm column. Column temperature was maintained at 25 °C. For elution, 50 mM H_3_PO_4_ (eluent *A*) and a 1:1 mixture of 100 mM phosphoric acid and acetonitrile (eluent *B*) were used at a flow rate of 1 ml min^−1^. The elution program started at 100% *A* and after 10 min of isocratic run, eluent *B* was increased linearly up to 100% in 5 min. Finally, the column was re-equilibrated with 100% *A* for 15 min. For fluorometric detection, the excitation and emission wavelengths were 290 nm and 395 nm, respectively^54^. Pure PL, PLP, PM and PN were purchased from Sigma-Aldrich and used as standards. The quantification was based on the measurements of these compounds with known concentrations.

### Sample preparation for antioxidant measurements

Leaves were ground in liquid nitrogen and then homogenized in ice cold Na-phosphate buffer (50 mM, pH 7.0) containing 1 mM EDTA. Samples for the ascorbate peroxidase measurements were made separately with the above grinding buffer containing 5 mM ascorbate as well. Leaf extracts were centrifuged (24,400 × g, 30 min, 4 °C) and supernatants were used for antioxidant capacity measurements. Protein contents were determined as described in Bradford’s protocol^55^.

### Antioxidant measurements on leaf samples

Superoxide dismutase (SOD, EC 1.15.1.1) activity measurements were carried out as described earlier^36^, based on the inhibition of nitroblue tetrazolium (NBT) reduction by xanthine - xanthine-oxidase generated superoxide anions, and results were expressed as U SOD mg^−1^ protein.

Class III peroxidase (POD, EC 1.11.1.7) activity was measured via the oxidation of ABTS (2,2′-azino-bis(3-ethylbenzothiazoline-6-sulphonic acid))^56^ that was found to be the most effective general POD substrate for UV-treated samples^57^. The colour change was detected by Multiskan FC plate reader (Thermo Fischer Scientific, Shanghai, China) at 651 nm and POD activities were given as U POD mg^−1^ protein.

Ascorbate peroxidase (APX, EC 1.11.1.11) activities were measured according to Nakano & Asada^58^, following ascorbate oxidation as decrease in absorbance at 295 nm with a spectrophotometer (Shimadzu UV-1800, Shimadzu Corporation, Tokyo, Japan). The reagent solution contained 0.5 mM ascorbic acid, 1 mM H_2_O_2_ and 1 mM EDTA in a Na-phosphate buffer (50 mM, pH 7.0) plus leaf samples. Values were corrected for the APX independent, direct oxidation of H_2_O_2_. Enzyme activities were described as U APX mg^−1^ protein.

Catalase (CAT, EC 1.11.1.6) activity was measured as described in Aebi *et al*.^59^, by following the decrease in H_2_O_2_ concentration as 240 nm absorbance for 70 seconds (measured at every second). The assay contained 18.6 mM H_2_O_2_ and 1 mM EDTA in 50 mM Na-phosphate buffer (pH 7.0) and the reaction was started by adding the leaf sample. Activity was expressed as U CAT mg^−1^ protein.

Glutathione peroxidase (GPX, EC 1.11.1.9) activity was determined by following NADPH oxidation at 340 nm according to Lawrence & Burk^60^. The reaction mixture contained 1 mM EDTA, 0.2 mM NADPH, 1 mM NaN_3_, 1 mM reduced glutathione and 1 U mL^−1^ glutathione reductase in 50 mM potassium phosphate buffer (pH 7.0) and the reaction was started by adding 0.25 mM H_2_O_2_. Following this, absorbance at 340 nm was measured (every second) for 4 min. Enzyme activity was determined as U GPX g^−1^ protein.

Hydroxyl radical (^•^OH) scavenging capacity was assessed via measuring the inhibition of the oxidation of terephthalic acid (TPA) to fluorescent hydroxyterephthalate (HTPA) by ^•^OH from the Fenton reaction^61^ in a Hitachi F-7000 spectrofluorimeter (Hitachi High-Technologies, Tokyo, Japan) with excitation at 315 nm and emission at 420 nm. The method is based on the fact that the antioxidant containing leaf samples can delay the ^•^OH-driven formation of HTPA. Hydroxyl radical antioxidant capacities were characterized by the amounts of plant samples needed to decrease HTPA fluorescence by 50%^62^ and were given as µM Trolox (6-Hydroxy-2,5,7,8-tetramethylchroman-2-carboxylic acid) equivalent g^−1^ leaf fresh weight.

### ROS specific antioxidant capacities of vitamin B_6_

Singlet oxygen (^1^O_2_) scavenging was determined by the ability of B_6_ vitamers to decrease the oxidation of DPBF (1,3-diphenylisobenzofuran) by ^1^O_2_^63^. As the ^1^O_2_ source we used methylene blue (MB) that was irradiated with 50 μmol m^−2^ s^−1^ red light (600–650 nm) for 1 minute. 1 mL of reaction mixture contained 20 μM MB and 100 μM DPBF in 60:40 v/v methanol/water. Oxidation of DPBF caused a decrease in absorbance which was followed at 410 nm using a Shimadzu UV-1800 spectrophotometer (Shimadzu Corporation, Kyoto, Japan). ^1^O_2_ scavenging abilities of B_6_ vitamers were assessed based on their ability to lessen the decrease of 410 nm absorbance and were presented as μM Trolox (6-hydroxy-2,5,7,8-tetramethylchroman-2-carboxylic acid) equivalents.

Superoxide anion (O_2_^•−^) antioxidant ability of vitamin B_6_ was characterized via a slightly modified assay described by Majer *et al*.^36^. B_6_ vitamers can inhibit the superoxide-induced reduction of NBT (nitro blue tetrazolium) to formazan. The reaction mixture contained 0.3 mM xanthine, 0.3 mM EDTA in 50 mM K-phosphate buffer (pH 7.2) and the reaction was started by adding 0.015 U xanthine oxidase. Formazan production was measured as absorbance change at 540 nm in a plate reader. Results were expressed as μM Trolox equivalents.

Hydrogen peroxide (H_2_O_2_) neutralization were measured using 3–3′-diaminobenzidine (DAB)^64^. 280 μM DAB and 400 μM H_2_O_2_ were dissolved in 50 mM Na-phosphate buffer (pH 7.0) and the reaction was started by adding 100 μU horseradish peroxidase and DAB oxidation was measured at 500 nm in a spectrophotometer. The H_2_O_2_ scavenging ability of B_6_ vitamers were defined as μM Trolox equivalents.

Hydroxyl radical (^•^OH) scavenging capacities were measured using a modification of the TBARS (thiobarbituric acid reactive substances) assay^65^. Such an indirect approach was necessary, because all B_6_ vitamers fluoresce upon UV excitation preventing the use of the more direct, terephthalate based method^61^ that was applied for characterizing leaf extracts. The assay contained 0.1 mM FeSO_4_, 1 mM EDTA, 0.25 mM ascorbate, 1 mM H_2_O_2_ and 3 mM deoxyribose in 20 mM potassium-phosphate buffer (pH 7.4). Hydroxyl radicals generated in a Fenton reaction oxidize deoxyribose yielding products that form a pink chromogen upon incubation with 0.1 w/v % thiobarbituric acid at 40 °C for 30 min in an 8% TCA solution. Added B_6_ vitamers competed with deoxyribose for the hydroxyl radicals and thus decreased chromogen formation that was followed as 540 nm absorbance using a plate reader. Results were expressed as μM Trolox equivalents.

### Statistics

All treatment groups contained 8 plants of each genotype as biological replicates that were assayed separately to form one (n = 8) data set. ROS reactivity measurements of pure vitamers, where no leaf material was used, were carried out with 6–8 technical repetitions to calculate one mean value. For each variable, differences between means were compared with two-sample Student’s t-tests and significantly different (*p* < 0.05) means are marked with either different letters (in graphs) or different symbols (in tables).
